# Visual Function Score: A New Clinical Tool to Assess Visual Function and Detect Visual Disorders in Children

**DOI:** 10.3389/fped.2022.868974

**Published:** 2022-04-26

**Authors:** Sabrina Signorini, Antonella Luparia, Giulia Cappagli, Eleonora Perotto, Mauro Antonini, Federica Morelli, Giorgia Aprile, Elena Ballante, Silvia Figini, Renato Borgatti, VFS Study Group

**Affiliations:** ^1^Developmental Neuro-ophthalmology Unit, IRCCS Mondino Foundation, Pavia, Italy; ^2^Child Neuropsychiatry Unit, IRCCS Mondino Foundation, Pavia, Italy; ^3^Department of Brain and Behavioural Sciences, University of Pavia, Pavia, Italy; ^4^BioData Science Center, IRCCS Mondino Foundation, Pavia, Italy; ^5^Department of Political and Social Sciences, University of Pavia, Pavia, Italy

**Keywords:** visual function, visual impairment, development, functional vision, rehabilitation, environmental adaptation

## Abstract

**Introduction:**

A comprehensive assessment of visual functioning at an early age is important not only for identifying and defining visual impairment but also for planning personalized rehabilitation programs based on the visual diagnosis. Since existing tools to evaluate visual functioning present some important limitations (e.g., they are based on qualitative reports, they do not take into account environmental adaptations of visual testing or they have not been formally validated as clinical instruments), the present work has the main aim to propose a new clinical tool (*Visual Function Score, VFS*) to detect and define visual disorders at an early age.

**Methods:**

The Visual Function Score was administered to one hundred visually impaired children (age range 4 months to 17.75 years old) in the form of a professional-reported protocol for a total of 51 items, each of which is assigned a score from 1 to 9 (or from 0 to 9 in some specific cases). The VFS produces three sub-scores and a global score (from 0 to 100), resulting in a quantitative evaluation of visual functioning.

**Results:**

The VFS can detect the well-known differences between different types of visual impairment (cerebral, oculomotor, and peripheral or grouped as central and peripheral) and takes into account different environments in the definition of a quantitative score of visual functioning.

**Discussion:**

Overall, the use of a quantitative tool to evaluate visual functions and functional vision such as the VFS would be fundamental to monitor the progresses of patients over time in response to rehabilitation interventions.

## Introduction

Visual impairment (VI) may have potential negative impact on children's development, daily living and education, as well as on the quality of their own life and their families' (1–4). The early diagnosis of VI and a comprehensive neuro-ophthalmological assessment also based on functional competencies is particularly relevant in the clinical context also for the purpose of planning and monitoring adequate rehabilitation interventions and should be based on a multidisciplinary clinical evaluation (5, 6). A global assessment of visual profile should include (i) a careful, structured clinical anamnesis, (ii) an ophthalmologic exam to assess the eye structures' status, and (iii) a clinical examination of the different *visual functions* (i.e., how the *visual system* functions). Moreover, it should also rely on a *functional vision* evaluation [i.e., how the *child* functions in vision-related activities, such as orientation and mobility, daily living skills, communication, and sustained vision tasks; (7, 8)]. Nonetheless, only rarely the assessment of vision relies on a comprehensive functional evaluation of visual competencies in the clinical practice (9–12). Several tools assess single visual functions (e.g., visual acuity) or functional vision even in very young children (13–16), while very few clinical instruments are based on a comprehensive assessment of both these aspects. Self-report questionnaires [e.g., the Cardiff Visual Ability Questionnaire for Children (17), the Impact of Vision Impairment for Children (18), the LV Prasad-Functional Vision Questionnaire (19), and the Children's Visual Function Questionnaire (20)] explore different domains of children's everyday life and have the merit to be based on information retrieved during focus groups with visually impaired children, providing a reliable description of the impact of VI on personal adaptive functioning. Nonetheless, they assess only general everyday competencies such as mobility, personality, family and school life (i.e., *functional vision* areas), but not visual functions. The Flemish CVI questionnaire (21–23) specifically assesses visuo-perceptual impairments in children aged 3–6 years. The Visual Function Classification System (VFCS) for children with cerebral palsy (24) describes children's visual profiles focusing on their visual abilities in daily life, proving useful information not only for the clinical settings but also to improve communication between healthcare professionals and families. The ABCDEFV battery provides an integrated evaluation of the child's visual functioning and his development stage (25). Furthermore, some batteries have been developed to assess visual function in specific populations, such as newborns at risk of visual and neurological abnormalities [e.g., the battery proposed by Ricci et al. (26), the Neonatal Behavioral Assessment Scale—NBAS (27), the Neonatal Assessment Visual European Grid—NAVEG (28)] or children with complex disabilities [e.g., the Bradford Visual Function Box—BVFB (29)]. Recently, several authors proposed rating scales and score system to quantify performances in visual functions. Some of these have been developed to assess specific components of visual function, such as ocular motility [e.g., the Ocular Motor Score—OMS (30) or the “oculomotor disorders” section of the International Cooperative Ataxia Rating Scale—ICARS (31)]. Besides their specific limitations, the above-mentioned tools present some general clinical limitations, which may lead to a lack of standardization in their use. Firstly, none of them takes into account environmental conditions that might facilitate visual performance. For instance, they do not include adaptations of testing materials or setting, which are an important aspect in the definition of the functional profile of a child (24). Secondly, some of these tools are inappropriate for use with children, since they include items related to adult-like activities (e.g., reading the newspaper). Thirdly, the majority of the tests and questionnaires refer to specific populations (e.g., newborns) or competences (e.g., ocular motility). Such evidences indicate that the development and validation of an assessment scale specifically designed to evaluate all aspects of visual functioning [i.e., the performance of components of the visual system (32)] in pediatric populations would be crucial. Such evaluation should be carried out regardless of the underlying clinical condition, as it has been proposed for different symptoms in the case of the ICARS (31). In the present study, we propose a new comprehensive tool designed to quantitatively assess children's visual functioning. The tool would allow to: (a) define children's visual function profile with its strengths and setbacks, (b) plan re-habilitation goals and training, (c) measure re-habilitation outcomes. The novelty of the tool is that it takes into account the environmental conditions that might facilitate visual performance, thus providing an idea about the impact of adaptations on the child's functional vision.

## Methods

### The Visual Function Score

The VFS is based on the idea that vision has three main components (33) that can be isolated and assessed separately: the component “seeing” refers to tasks related to the primary visual pathways (visual acuity, visual field and contrast sensitivity); the component “looking” concerns the oculomotor system and its function (e.g., saccades); the component “understanding” relates to the dorsal and ventral streams, thus to visuo-cognitive (gnosic and spatial) abilities. The VFS is composed of four sections (see Supplementary Materials) and evaluates the first two components of vision, taking into consideration also purely ocular aspects that could play a role in influencing these components (such as refraction, fundus oculi, and anterior segment).

**Figure 1:**
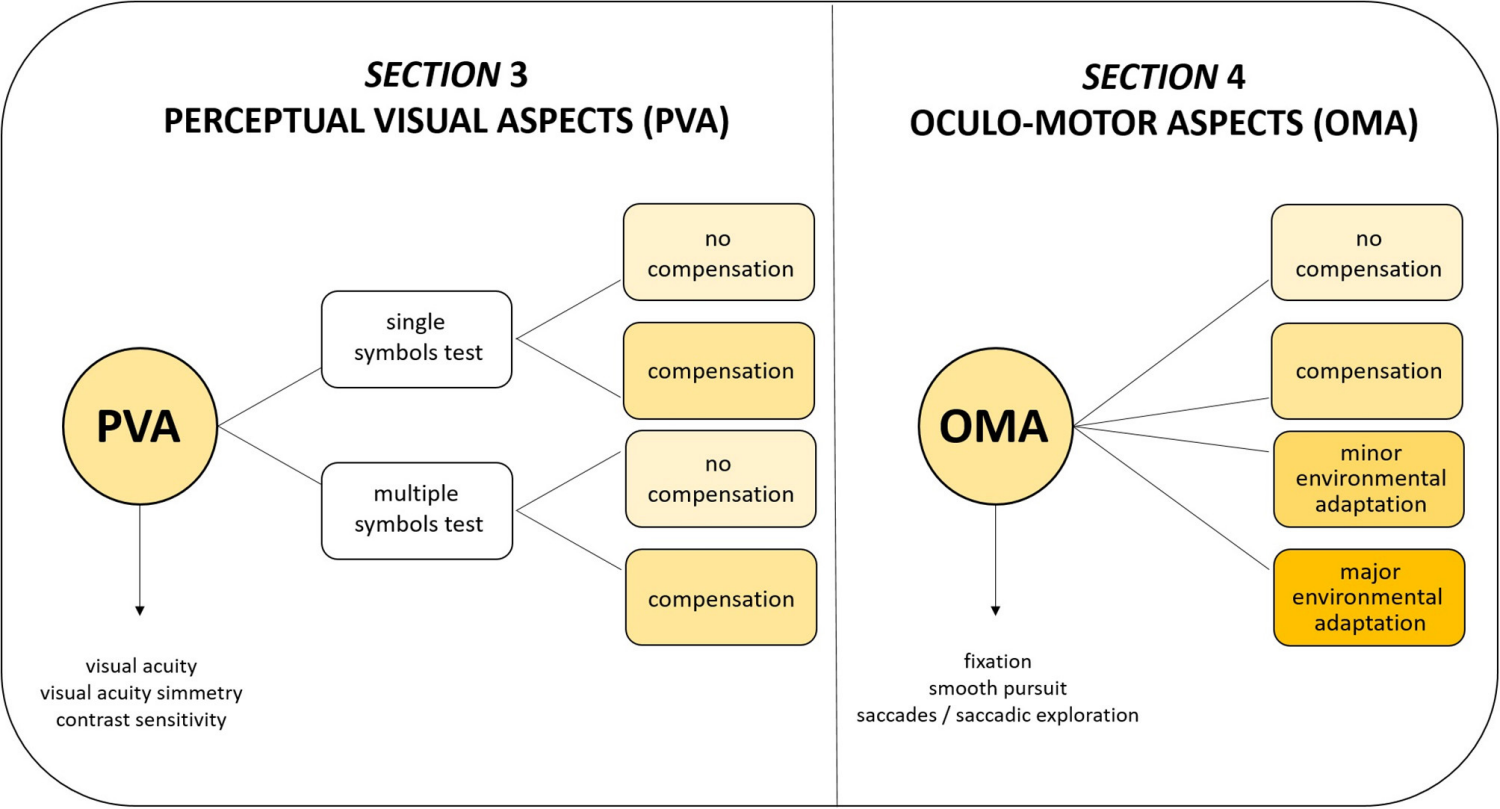
Visual Function Score conception.

The VFS takes into consideration the use of compensatory strategies (such as head, eye, and body position) and reduced visual crowding (i.e., use of single symbols for testing visual acuity and contrast sensitivity) for Perceptual Visual Aspects (PVA, Section 3). For Oculo-motor Aspects (OMA, Section 4), four level of adaptations have been considered (see Figure 1):

- Level A (no compensation): no self-adopted compensatory strategy nor environmental adaptation of setting (a normal object is presented in a neutral setting).
- Level B (compensation): spontaneous use of compensatory strategy (without environmental adaptation).
- Level C (minor environmental adaptation): minor environmental adaptation (for example, adjustment of distance or use of high contrast and structured patterns).
- Level D (major environmental adaptation): major environmental adaptation for example, use of illuminated target in adjusted lighting conditions and multisensory setting.

### Administration and Score Calculation

The VFS was conceived by a multi-disciplinary team with long-term experience in childhood visual disorders as a tool that can be administered by a single professional at a time and is based on the clinical protocol used in our Center (34) with the help of the standardized tests according to the age. For some items, we specify the age-limit for the administration. The clinician is asked to assign a score to each item, according to the instructions listed in the template (see Supplementary Materials). The total score (TOT) of a patient is defined as the weighted sum of the 51 items recorded in section 2 (Ocular-Visual Aspects or OVA), 3 (PVA), and 4 (OMA) which is normalized to obtain a score from 0 to 100 (calculated as $\frac{total\;score-minimum\;of\;total\;score}{maximum\;of\;total\;score}\;*100$). Moreover, three additional subscores can be obtained (OV, ocular-visual; PV, perceptual-visual; OM, ocular-motor), all normalized to obtain a score from 0 to 100. We specify that the same scoring system has been used also for parameters that can be quantified according to normative values (e.g., visual acuity), to allow a comparison between the other visual functions.

### Participants

One-hundred patients were retrospectively enrolled from the Center of Child Neuro-ophthalmology, IRCCS Mondino Foundation (Pavia, Italy). The tool was administered by a professional clinician with a long-term experience with visually impaired children. The total sample comprised 61 males and 39 females, aged between 4 months and 17.75 years (mean age 8 ± 4.37 years) with different diagnoses causing the VI (for the diagnosis distribution, see Supplementary Figure 1). The study protocol was approved by the institutional ethics committee and written consent was acquired from enrolled children's caregivers.

Patients were grouped according to two criteria. The first criterion allows to group patients based on involvement of visual subcomponents: retro-geniculostriate primary visual pathway (Cerebral Visual Impairment, CVI), oculo-motor system (Oculo-Motor Visual Impairment, OMVI), and retino-pre-geniculostriate primary visual pathways that is eye and optic nerve (Peripheral Visual Impairment, PVI). According to the first criterion, 28% presented CVI, 16% OMVI, and 56% PVI. The second criterion allows to group patients based on the nature of the deficit according to the involvement of central (visual brain and oculomotor system) or peripheral visual pathways (e.g., eye, cranial nerves, ocular muscles). According to the second criterion, 52% received a diagnosis of peripheral (i.e., ocular) condition, and 48% of a central (i.e., neurologic) condition, including central oculomotor disturbances.

Patients enrolled in this study protocol received a single visual disorder diagnosis but could also present a combined disorder [e.g., retinal dystrophy and oculomotor apraxia in Joubert syndrome (35)]. Neuromotor deficit was also considered as grouping factor, when present, to consider its impact on visual performance; specifically, a total number of 40 subjects were included in the study presenting hemiplegia (12.5%), diplegia (27.5%), quadriplegia (25%), and cerebellar syndrome (35%).

### Statistical Analysis

The analysis focused on three main aspects: (1) the discriminant power of the tool to differentiate different types of visual impairments; (2) the discriminant power of the tool to characterize neuromotor deficits; (3) the advantage of compensation and adaptations strategies on visual functioning. Numerical variables are described as minimum, maximum, median, mean, and standard deviation, categorical variables as row count and percentage. The significance level was set to 0.05, but also *p*-values between 0.05 and 0.1 are reported. Since the assumption of normality was not met, non-parametric methods were applied. Kruskal–Wallis one-way analysis of variance (non-parametric ANOVA) was used to compare groups in terms of score and subscores. Dunn tests were performed as *post-hoc* test for pairwise comparisons and the *p*-values were corrected according to Bonferroni criterion. In order to compare of groups and environments (between- and within-groups factors), Wald tests are implemented. Pairwise comparisons are performed using Dunn tests (independent groups) and Durbin-Conover test (paired groups), the *p*-values are corrected with Bonferroni method.

## Results

### Discriminant Power Related to Visual Impairments

**Figure 2:**
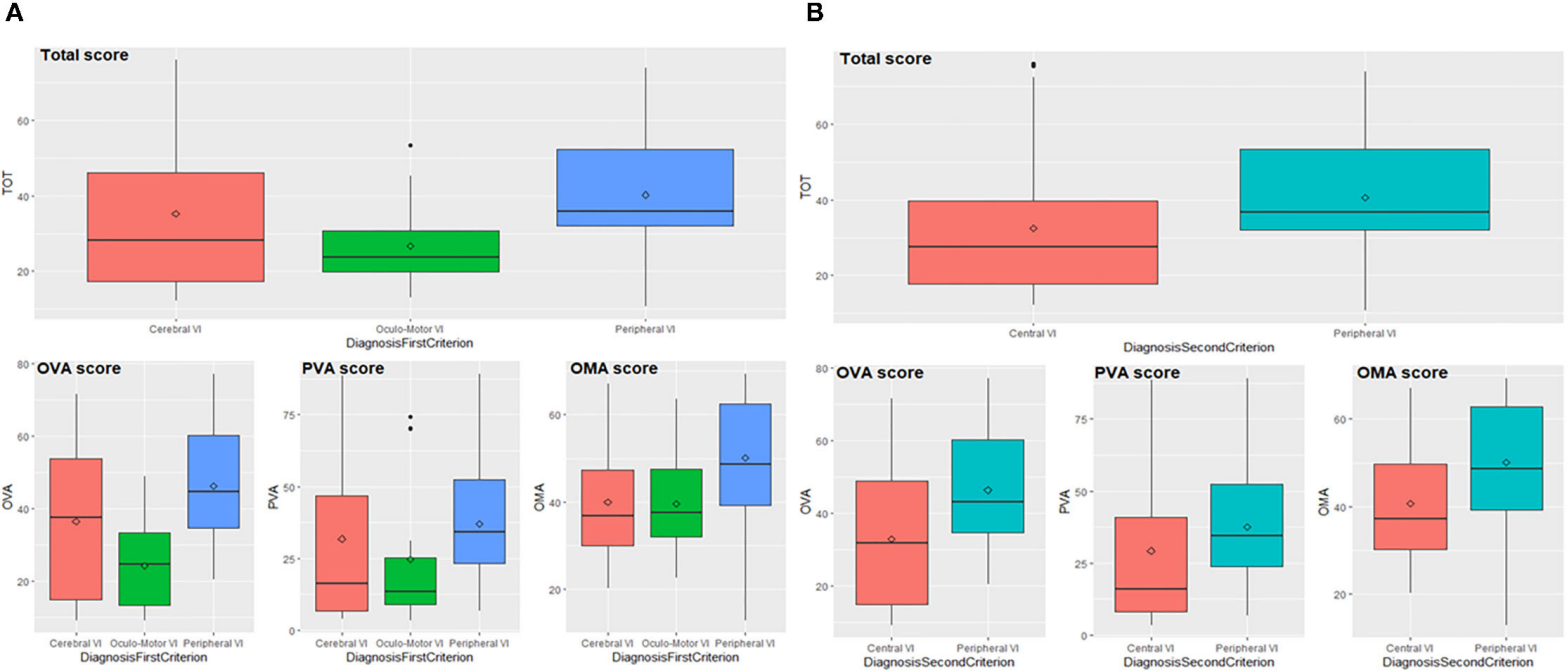
Total score and subscores according to first **(A)** and second **(B)** diagnostic criterion. OVA, Ocular-Visual Aspects; PVA, Perceptual Visual Aspects; OMA, Oculo-Motor Aspects; VI, Visual Impairment.

We demonstrate that the VFS reflects the well-known differences between the types of visual impairment. The total score and subscores significantly differ in patients grouped according to the first criterion (*Cerebral* vs. *Oculo-Motor* vs. *Peripheral VI*) since *p*-values of the ANOVA-type analyses are lower than 0.01 (Figure 2A and Supplementary Table 1).

Pairwise comparisons indicate that patients with PVI and OMVI differ in their total score and subscores: the first scored higher compared to the second group (total score *p* = 0.002, OVA *p* < 0.001, PVA *p* = 0.026, OMA *p* = 0.020). Neither total score and subscores can detect differences between patients with OMVI and CVI with a significance level of 0.05, but through the enlargement of the significance level to 0.1, subscore OVA results significantly different in the comparison of OMVI and CVI (*p* = 0.080), since the first implies lower values than the seconds. Patients with PVI and CVI significantly differ in their total score and subscore OMA (total score *p* = 0.045, OMA *p* = 0.003), while the *p*-values related to OVA and PVA subscores are slightly above the threshold (*p* = 0.077 and *p* = 0.052). PVI group scored higher compared to CVI group. Data analyses performed on patients grouped according to the second criterion (*Central* vs. *Peripheral VI*) indicate that total score and subscores significantly differ between groups (*p*-values lower than 0.01), as shown in Figure 2B (see also Supplementary Table 2). In fact, patients with peripheral impairment obtain higher values than patients with central impairment.

### Discriminant Power Related to Neuromotor Deficits

**Figure 3:**
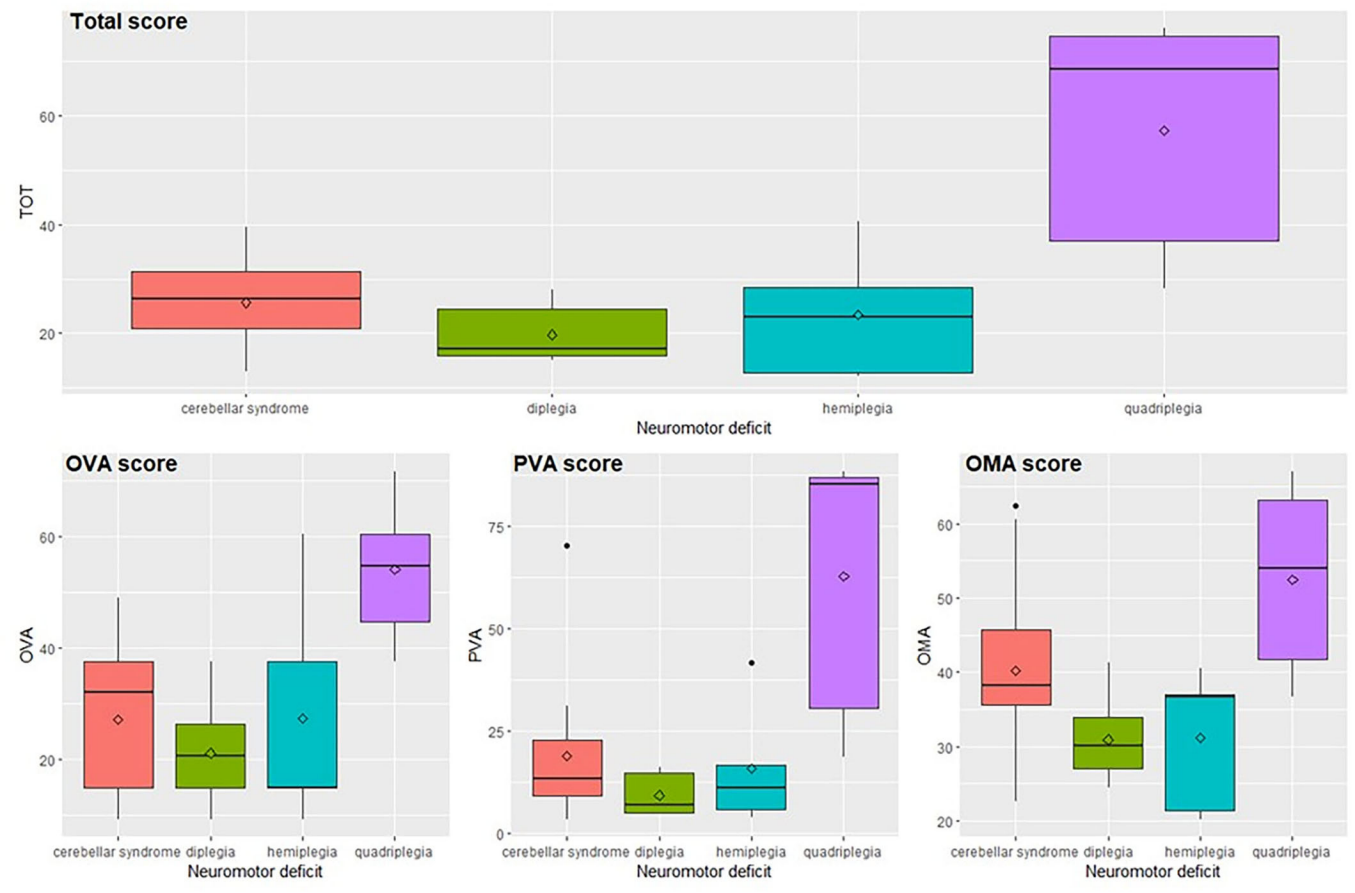
Total score and subscores in relation to the neuromotor deficit. OVA, Ocular-Visual Aspects; PVA, Perceptual Visual Aspects; OMA, Oculo-Motor Aspects.

Results related to neuromotor deficits (Figure 3 and Supplementary Table 3) indicate that the total score and subscores are significantly different among the neuromotor deficits taken into consideration (total score, OVA and PVA *p* < 0.001, OMA *p* = 0.002) according to the ANOVA-type analyses. The significant pairwise comparisons are quadriplegia vs. all other deficits where the values of subjects with quadriplegia obtain scores significantly higher than the other three categories (total score *p*-values lower than 0.025, OVA *p*-values lower than 0.045, PVA *p*-values lower than 0.040, OMA *p*-values lower than 0.035) with the only exception of the comparison of OMA score between quadriplegia and cerebellar syndrome where the difference is not significant.

### The Role of Compensatory and Environmental Adaptation Strategies

**Figure 4:**
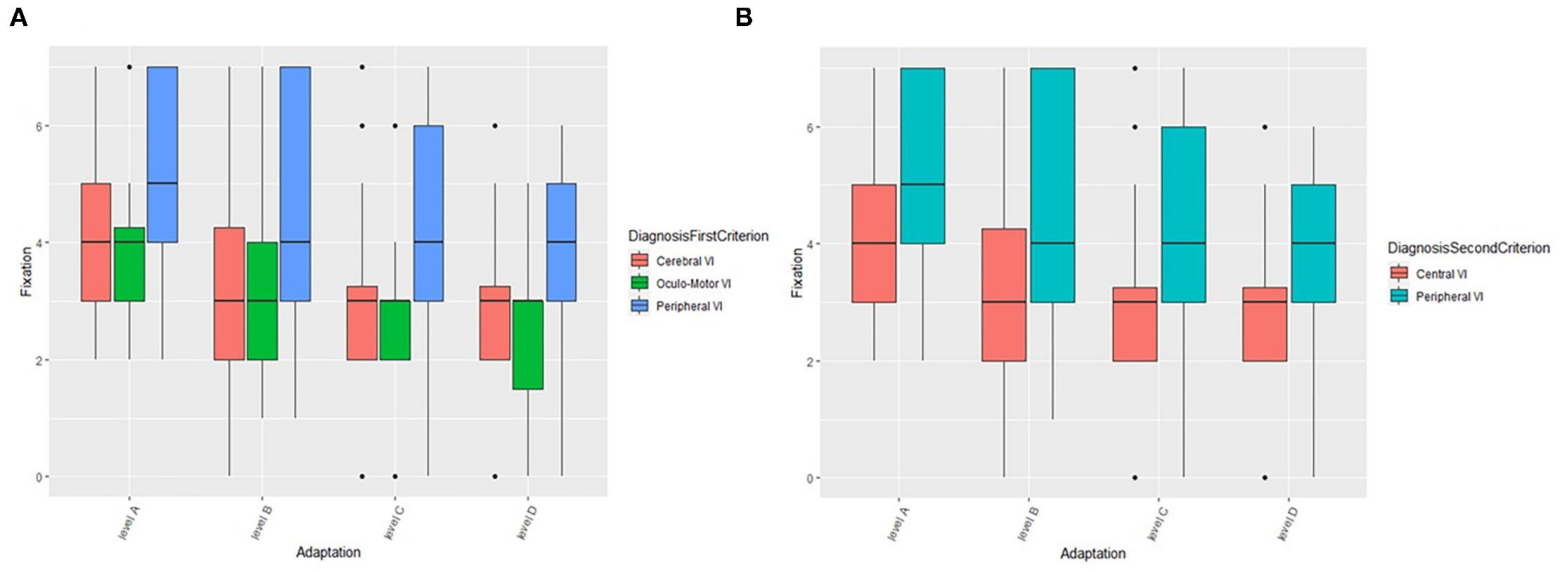
The role of compensation strategies and environmental adaptations on fixation according to the first **(A)** and second **(B)** criterion. VI, Visual Impairment.

In this section, we describe the role of compensatory and environmental adaptation strategies on fixation as an example, while results concerning other oculo-motor abilities (smooth pursuit and saccades) are presented in the Supplementary Materials. For what concerns fixation (Figure 4A), values are higher in natural setting without adaptations (level A) and decrease when self-adopted compensation and/or environmental adaptations are introduced (levels B, C, and D). More in detail, the fixation is significantly different between levels of adaptation (*F* = 453.3, *df* = 3, *p* < 0.001) and the pairwise comparisons confirm the result (*p*-values of pairwise comparisons are lower than 0.001). The same decreasing trend is showed if we considered the first criterion of grouping (*Cerebral* vs. *Oculo-Motor* vs. *Peripheral VI*), i.e., there is no statistical interaction between groups and adaptations (self-adopted/environmental), but PVI seems to be characterized by higher values in each environment. Fixation is significantly different between groups (*F* = 16.9, *df* = 2, *p* < 0.001). Moreover, pairwise groups comparisons are statistically significant between OMVI vs. PVI (*z* = 5.4, *p* < 0.001) and CVI vs. PVI (*z* = 6.1, *p* < 0.001), while there is no significant difference between CVI and OMVI. Patients with PVI shows higher values of fixation than CVI and OMVI patients, confirming the considerations made above. The same analysis performed on subjects grouped by the second criterion (*Central* vs. *Peripheral VI*) (Figure 4B) confirms the results. The Wald test shows that the fixation is significantly different between levels of adaptation (*F* = 824.1, *df* = 3, *p* < 0.001) and more specifically the fixation values are higher in natural setting (level A) and decrease when adaptations are introduced (levels B, C, and D). In fact, fixation values are significantly different among the level of adaptations (all *p*-values of pairwise comparison are lower than 0.001). The same decreasing trend between adaptation levels is showed when we applied the second criterion of grouping (*Central* vs. *Peripheral VI*), i.e., there is no statistical interaction between groups and environments, but PVI is characterized by higher value in each environment. This result is confirmed by the significance of the grouping factor defined as second criterion (*F* = 16.2, *df* = 1, *p* < 0.001).

## Discussion

The early diagnosis of visual impairments and the comprehensive assessment of visual functioning in all its aspects (ocular, perceptual, and oculo-motor) are crucial to plan personalized re-habilitation interventions based on patients' visual profile. Moreover, a periodic assessment of visual functions to monitor visual and clinical profile allows to constantly adapt intervention strategies over time (36). For these reasons, a comprehensive assessment of visual functioning would be fundamental to identify areas of strength and frailty of patients from an early age. To our knowledge, while some visual functions (e.g., visual acuity or contrast sensitivity) can be quantified with age-specific tests, many competencies (e.g., oculo-motor functions) are usually evaluated qualitatively, especially in children with comorbidities. Moreover, many of the available tools have not been adequately tested for validity and reliability and might present some limitations in detecting clinically relevant changes in visual performance over time. Finally, available clinical tests do not take into account the effect of compensatory strategies and environmental adaptations as facilitators, as would be suggested by recent studies sustaining the effectiveness of the Environmental Enrichment Approach on specific developmental competencies (36).

In the present work, we developed a new clinical tool (Visual Function Score—VFS) to quantitatively assess visual functioning in all its components from an early age and even in children with complex disabilities. The main novelties of the VFS are: (a) a comprehensive evaluation of different visual functions; (b) the quantification of the visual performance taking into account self-adopted compensatory strategies (i.e., spontaneously adopted by the child, as in case of head turn) and adaptations of visual environment (i.e., setting adaptation needed by the patient to carry out a specific task); (c) the attribution of a score to visual functions considering the use of different tests based on patients' age (e.g., grating and visual acuity), which widens the age range of administration. The adaptation of testing materials and setting allows to assess and identify any modifiable aspects of the child's vision. Visual environment adaptations (i.e., use of structured-pattern targets, regulation of room lighting, use of high-contrasted backgrounds or multisensory adapted settings) are in fact crucial to facilitate proactiveness in exploration, action and interaction, allowing the visually-impaired child to make the most of his residual and potential visual function. With this study, we demonstrated that the VFS allows to: (1) quantitatively measure subcomponents of visual functions or more functions referring to the same visual subsystem (e.g., all oculo-motor competencies), (2) monitor re-habilitation outcomes, and (3) define the most suitable re-habilitation setting based on the environmental adaptations needed according to the child's visual functioning.

The use of self-adopted compensatory strategies and environmental adaptations in a quantitative visual function score is crucial because it allows identifying the optimal characteristics to promote the use of visual potential both in the therapeutic setting and life contexts of the child. Moreover, a more correct sensorial experience can promote visual system maturation, with the support of cerebral plasticity, thus reducing the necessity of environmental adaptations over time. The VFS can be a powerful tool for diagnosing visual disorders because it permits to differentiate children's profiles according to a comprehensive set of abilities. Moreover, it can be adopted to evaluate rehabilitation outcomes because it gives a measure of the level of environmental adaptations needed by patients to perform the testing activities, presuming that for some conditions the more the child advances in the rehabilitation program the less he will use environmental adaptations. The assessment of validity, reliability, discriminant capacity, and inter-rater agreement of the tool was performed on this preliminary data with promising results, together with consistency with existing methods, comparing the proposed score with the VFCS (24). A statistically significant validation will be presented in a future work and is beyond the scope of this paper. The validated instrument will be made available online as an interactive interface accompanied by a detailed guide and didactic material. We believe that the VFS, together with other clinical tools such as self-report questionnaires for parents or instruments for specific populations (e.g., VFCS), would be a valuable tool for a comprehensive assessment including not only visual function, but also functional vision.

## Data Availability Statement

Original data are available on a repository at the link doi: 10.5281/zenodo.4973336.

## Ethics Statement

The studies involving human participants were reviewed and approved by Ethics Committee of Pavia Area, Fondazione IRCCS Policlinico San Matteo, Pavia (Italy). Written informed consent to participate in this study was provided by the participants' legal guardian/next of kin.

## Author Contributions

All authors conceived the study protocol and the rationale for the project. SS, AL, and EP coordinated recruitment of participants and data collection. AL, EP, and MA were directly involved in the evaluations, and contributed to data collection. SS, SF, and RB supervised the progress of the study. SF and EB performed data analyses. SS, GC, FM, GA, and EB wrote and revised the manuscript. RB and SF provided suggestions for the improvement of the manuscript. All authors contributed to the article and approved the submitted version.

## Funding

This research was supported by a grant from the Italian Ministry of Health (RC 2020).

## Conflict of Interest

The authors declare that the research was conducted in the absence of any commercial or financial relationships that could be construed as a potential conflict of interest.

## Publisher's Note

All claims expressed in this article are solely those of the authors and do not necessarily represent those of their affiliated organizations, or those of the publisher, the editors and the reviewers. Any product that may be evaluated in this article, or claim that may be made by its manufacturer, is not guaranteed or endorsed by the publisher.
