# Supplementary material for: Visual Function Score: A New Clinical Tool to Assess Visual Function and Detect Visual Disorders in Children
Source: Front Pediatr. 2022 Apr 26;10:868974. doi: 10.3389/fped.2022.868974 (PMC9087345; doi:10.3389/fped.2022.868974)
Supplement: Supplementary file 1 [file Data_Sheet_1.pdf]

Supplementary Material

1 Supplementary Figures and Tables

	Diagnosis	Min	Max	Median	Mean	SD	pvalue
**TOT**	Cerebral VI	12.1	76.1	28.2	32.2	22.0	0.0010
	Oculo-Motor VI	13.0	53.5	23.7	26.8	11.3	
	Peripheral VI	10.6	73.9	35.9	40.3	13.9	
**OVA**	Cerebral VI	9.2	71.6	37.6	36.5	19.8	<0.0001
	Oculo-Motor VI	9.2	48.9	24.8	24.3	13.1	
	Peripheral VI	20.6	77.3	44.7	46.3	14.3	
**PVA**	Cerebral VI	3.9	88.5	16.3	31.8	32.1	0.0072
	Oculo-Motor VI	3.4	74.3	13.4	24.6	24.5	
	Peripheral VI	6.6	89.3	34.0	37.1	18.8	
**OMA**	Cerebral VI	20.2	67.1	36.9	40.0	13.9	0.0008
	Oculo-Motor VI	22.6	63.6	37.5	39.6	12.1	
	Peripheral VI	12.9	69.4	48.7	50.0	13.8	

Supplementary Table 1: Total score and subscores according to first diagnostic criterion. OVA: Ocular-Visual Aspects; PVA: Perceptual Visual Aspects; OMA: Oculo-Motor Aspects; VI: Visual Impairment.

	Diagnosis	Min	Max	Median	Mean	SD	pvalue
**TOT**	Central VI	12.1	76.1	27.5	32.4	18.6	0.0005
	Peripheral VI	10.62	73.9	36.7	40.6	14.0	
**OVA**	Central VI	9.2	71.6	31.9	33.0	18.1	0.0003
	Peripheral VI	20.6	77.3	43.3	46.4	14.7	
**PVA**	Central VI	3.4	88.5	16.0	29.2	29.2	0.0013
	Peripheral VI	6.6	89.3	34.3	37.6	18.0	
**OMA**	Central VI	20.2	67.1	37.3	40.6	13.4	0.0004
	Peripheral VI	12.9	69.4	48.7	50.1	13.9	

Supplementary Table 2: Total score and subscores according to second diagnostic criterion. OVA: Ocular-Visual Aspects; PVA: Perceptual Visual Aspects; OMA: Oculo-Motor Aspects; VI: Visual Impairment.

	Diagnosis	Min	Max	Median	Mean	SD	pvalue
**TOT**	hemiplegia	12.1	40.6	22.9	23.4	11.9	0.0002
	diplegia	15.0	28.0	17.3	19.8	5.0	
	quadriplegia	28.3	76.1	68.5	57.3	20.6	
	cerebellar syndrome	13.0	39.5	26.3	25.6	7.9	
**OVA**	hemiplegia	9.2	60.3	14.9	27.4	21.4	0.0004
	diplegia	9.2	37.6	20.6	21.1	8.6	
	quadriplegia	37.6	71.6	54.6	54.0	11.2	
	cerebellar syndrome	9.2	48.9	31.9	27.1	14.1	
**PVA**	hemiplegia	3.9	41.6	11.2	15.8	15.2	0.0002
	diplegia	4.6	16.1	6.8	9.2	5.0	
	quadriplegia	18.7	88.5	85.4	62.9	31.5	
	cerebellar syndrome	3.4	70.4	13.4	18.9	16.8	
**OMA**	hemiplegia	20.2	40.4	36.7	31.1	9.6	0.0015
	diplegia	24.4	41.3	30.1	30.9	5.4	
	quadriplegia	36.7	67.1	54.0	52.4	12.0	
	cerebellar syndrome	22.6	62.4	38.2	40.2	12.0	

Supplementary Table 3: Total score and subscores in relation to neuromotor deficit. OVA: Ocular-Visual Aspects; PVA: Perceptual Visual Aspects; OMA: Oculo-Motor Aspects.

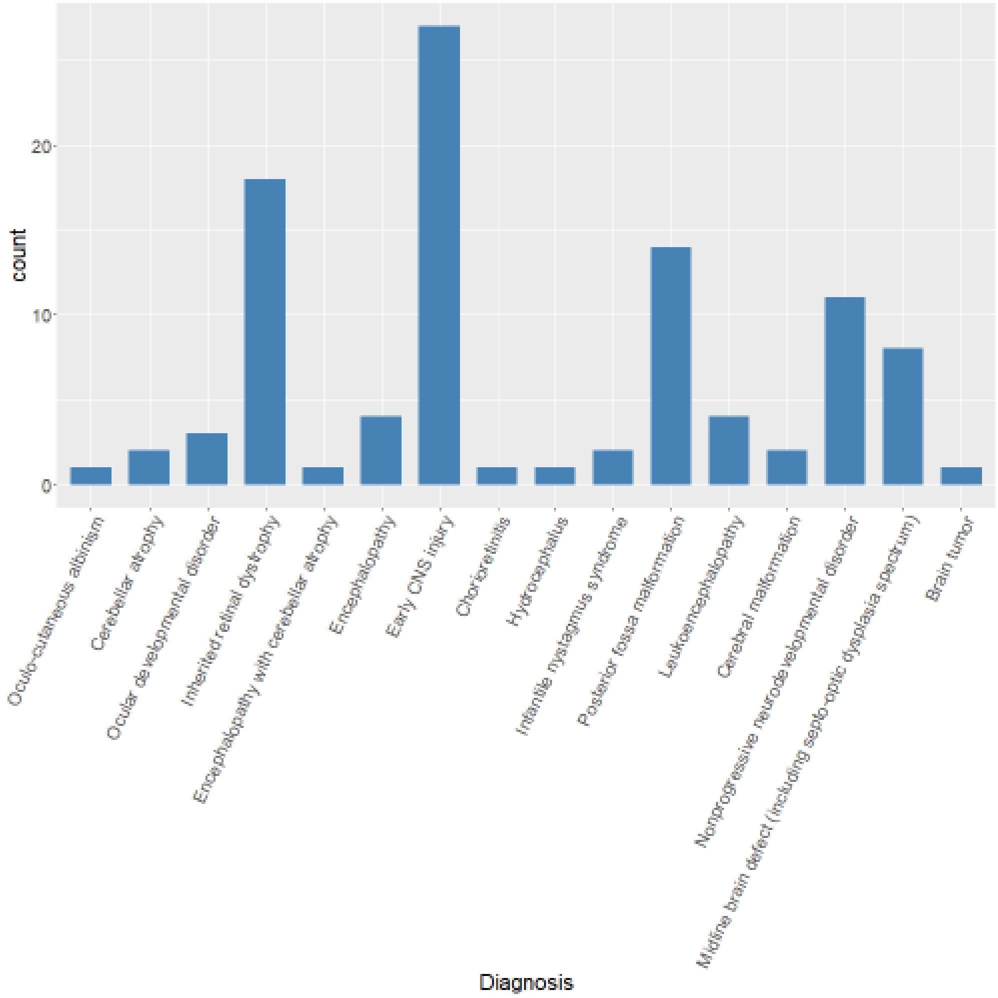

Supplementary Figure 1. Diagnosis distribution in the total sample of the study.

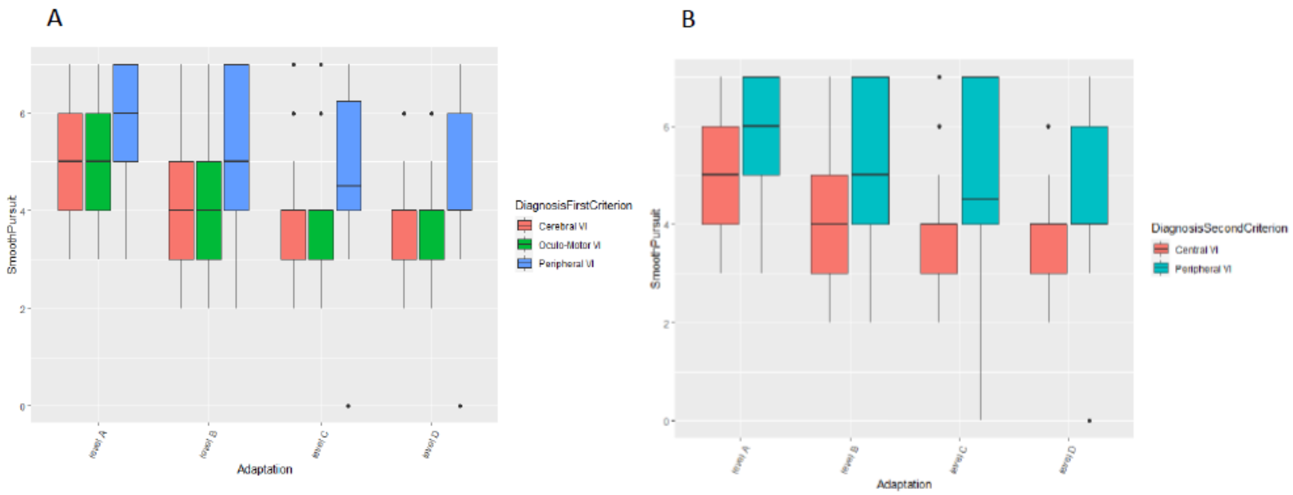

Supplementary Figure 2: The role of compensation strategies and environmental adaptations on Smooth Pursuit according to the first (A) and second (B) criterion. VI: Visual Impairment.

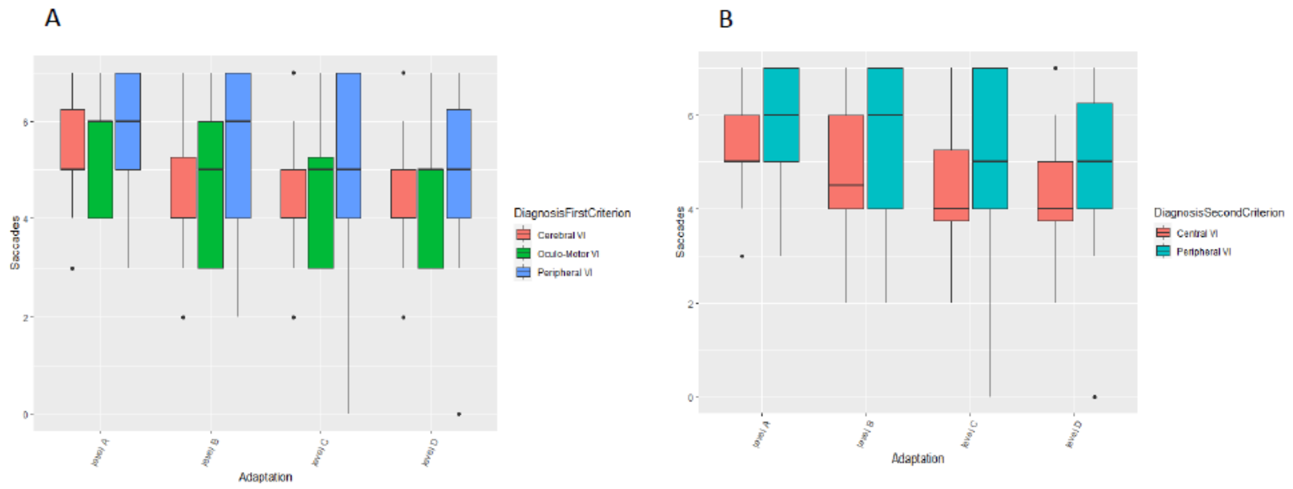

Supplementary Figure 3: The role of compensation strategies and environmental adaptations on Saccades according to the first (A) and second (B) criterion. VI: Visual Impairment
