# Supplementary material for: Visual Function Score: A New Clinical Tool to Assess Visual Function and Detect Visual Disorders in Children
Source: Front Pediatr. 2022 Apr 26;10:868974. doi: 10.3389/fped.2022.868974 (PMC9087345; doi:10.3389/fped.2022.868974)
Supplement: Supplementary file 2 [file Data_Sheet_2.pdf]

## VISUAL FUNCTION SCORE – VFS

The administration must be carried out in a setting of adequate collaboration and behavioral regulation, taking into account the age of the child, the general clinical picture and the attention span. Sections 2, 3 and 4 represent the core component of the VFS; section 1 (“Demographic and clinical details”), although not included within the final score, provides a better understanding of the results highlighting interactions between vision and general clinical picture.

### SECTION 1 DEMOGRAPHIC AND CLINICAL DETAILS

| | |
|---|---|
| **Name and Surname** | |
| **Date of birth** | |
| **Gender** | |
| **Date of assessment** | |
| **Age of assessment** | |
| **Main diagnosis** | |
| **Neurophtalmological diagnosis** | |

| | |
|---|---|
| **Neuromotor disorder** | 0. absent<br>1. dyspraxia / motor coordination impairment or aspecific features such as hypotonia<br>2. hemiplegia<br>3. diplegia<br>4. quadriplegia<br>5. pyramidal syndrome (not CP)<br>6. cerebellar syndrome<br>7. extrapiramidal syndrome<br>8. progressive disorder<br>9. not defined neuromotor disorder |
| **Psychomotor delay (<5 yo)** | 0. absent<br>1. present |
| **Intellectual disability** | 0. absent<br>1. mild<br>2. moderate<br>3. severe<br>4. unspecified but present<br>5. not testable (for age, collaboration, etc) |
| **Epilepsy** | 0. absent<br>1. EEG abnormalities<br>2. present (drug-responsive)<br>3. present (drug-resistant) |
| **Posture (preferential)** | 0. indifferent<br>1. conditioned by neuromotor disorder (no aid necessary) |

| | |
|---|---|
| | 2. aid necessary |
| **Corrective lenses** | 0. not necessary (emmetropia)<br>1. necessary<br>2. necessary, but no compliance<br>3. not prescribed for the severe of visual picture<br>4. refraction not testable |
| **Visual Evoked Potentials** | 0. Normal<br>1. Mild alteration (one eye)<br>2. Mild alteration (both eyes)<br>3. Moderate to severe alteration (one eye)<br>4. Moderate to severe alteration (both eyes)<br>5. Estinguished (one eye)<br>6. Estinguished (both eyes)<br>7. Not testable due to lack of cooperation or a too severe clinical picture<br>8. Missing data |
| **Electroretinogram** | 0. Normal<br>1. Mild alteration (one eye)<br>2. Mild alteration (both eyes)<br>3. Moderate to severe alteration (one eye)<br>4. Moderate to severe alteration (both eyes)<br>5. Estinguished (one eye)<br>6. Estinguished (both eyes)<br>7. Not testable due to lack of cooperation or a too severe clinical picture<br>8. Missing data |

## SECTION 2 OCULAR-VISUAL ASPECTS

**Refraction**

| | | |
|---|---|---|
| Emmetropia | 1 | |
| Low (1-3 diopters) refractive error (one eye) | 2 | |
| Low (1-3 diopters) refractive error (both eyes) | 3 | |
| Moderate (4-6 diopters) refractive error (one eye) | 4 | |
| Moderate (4-6 diopters) refractive error (both eyes) | 5 | |
| High (>6 diopters) refractive error (one eye) | 6 | |
| High (>6 diopters) refractive error (both eyes) | 7 | |
| Not testable due to lack of cooperation or a too severe clinical picture | 9 | |
| Missing data | 99 | |

**Fundus oculi**

| | | |
|---|---|---|
| Normal | 1 | |
| Mild alteration of optic nerve head/retinal vessels/retina (one eye) | 2 | |
| Mild alteration of optic nerve head/retinal vessels/retina (both eyes) | 3 | |
| Moderate to severe non-progressive alteration of optic nerve head/retina (one eye) | 4 | |
| Moderate to severe non-progressive alteration of optic nerve head/retina (both eyes) | 5 | |
| Moderate to severe progressive alteration of optic nerve head/retina (one eye) | 6 | |
| Moderate to severe progressive alteration of optic nerve head/retina (both eyes) | 7 | |
| Not testable due to lack of cooperation or a too severe clinical picture | 9 | |
| Missing data | 99 | |

**Anterior Segment**

| | | |
|---|---|---|
| Normal | 1 | |
| Altered (one eye) | 2 | |
| Altered (both eyes) | 3 | |
| Not testable due to lack of cooperation or a too severe clinical picture | 9 | |
| Missing data | 99 | |

## SECTION 3 PERCEPTUAL-VISUAL ASPECTS

**Visual acuity (VA)**

| | | | | | |
|---|---|---|---|---|---|
| Distance Visual Acuity (3-5 meters) | Multiple Symbols (Line Tests) | No compensatory strategies | Not testable for age (<4yo) | 00 | |
| | | | Normal (>7/10) (in this case sign as 'not necessary'* the next levels for distance VA) | 1 | |
| | | | Near-normal (>3 and ≤7/10) | 2 | |
| | | | Mild low vision (> 2 and ≤3/10) | 3 | |
| | | | Moderate low vision (> 1 and ≤2/10) | 4 | |
| | | | Severe low vision (> 0.05 and ≤1/10) | 5 | |
| | | | Partial blindness (≤0.05/10) | 6 | |
| | | | Total blindness | 7 | |
| | | | Not testable due to lack of cooperation or a too severe clinical picture | 9 | |
| | | | Missing data | 99 | |
| | | Compensatory stategies | Not necessary* | 0 | |
| | | | Not testable for age (<4yo) | 00 | |
| | | | Normal (>7/10) (in this case sign as 'not necessary'* the next levels for distance VA) | 1 | |
| | | | Near-normal (>3 and ≤7/10) | 2 | |
| | | | Mild low vision (> 2 and ≤3/10) | 3 | |
| | | | Moderate low vision (> 1 and ≤2/10) | 4 | |
| | | | Severe low vision (> 0.05 and ≤1/10) | 5 | |
| | | | Partial blindness (≤0.05/10) | 6 | |
| | | | Total blindness | 7 | |
| | | | Not testable due to lack of cooperation or a too severe clinical picture | 9 | |
| | | | Missing data | 99 | |

| | | | | | |
|---|---|---|---|---|---|
| Distance Visual Acuity (3-5 meters) | Single Symbols Test | No compensatory strategies | Not necessary* | 0 | |
| | | | Not testable for age (<4yo) | 00 | |
| | | | Normal (>7/10) (in this case sign as 'not necessary'* the next levels for distance VA) | 1 | |
| | | | Near-normal (>3 and ≤7/10) | 2 | |
| | | | Mild low vision (> 2 and ≤3/10) | 3 | |
| | | | Moderate low vision (> 1 and ≤2/10) | 4 | |
| | | | Severe low vision (> 0.05 and ≤1/10) | 5 | |
| | | | Partial blindness (≤0.05/10) | 6 | |
| | | | Total blindness | 7 | |
| | | | Not testable due to lack of cooperation or a too severe clinical picture | 9 | |
| | | | Missing data | 99 | |
| | | Compensatory stategies | Not necessary* | 0 | |
| | | | Not testable for age (<4yo) | 00 | |
| | | | Normal (>7/10) | 1 | |
| | | | Near-normal (>3 and ≤7/10) | 2 | |
| | | | Mild low vision (> 2 and ≤3/10) | 3 | |
| | | | Moderate low vision (> 1 and ≤2/10) | 4 | |
| | | | Severe low vision (> 0.05 and ≤1/10) | 5 | |
| | | | Partial blindness (≤0.05/10) | 6 | |
| | | | Total blindness | 7 | |
| | | | Not testable due to lack of cooperation or a too severe clinical picture | 9 | |
| | | | Missing data | 99 | |

| | | | | | |
|---|---|---|---|---|---|
| Near Visual Acuity | Multiple Symbols (Line Tests) | No compensatory strategies | Not testable for age (<2.5yo) | 00 | |
| | | | Normal (>7/10) (in this case sign as 'not necessary'* the next levels for near VA) | 1 | |
| | | | Near-normal (>3 and ≤7/10) | 2 | |

| | | | | | |
|---|---|---|---|---|---|
| | | | Mild low vision (> 2 and ≤3/10) | 3 | |
| | | | Moderate low vision (> 1 and ≤2/10) | 4 | |
| | | | Severe low vision (> 0.05 and ≤1/10) | 5 | |
| | | | Partial blindness (≤0.05/10) | 6 | |
| | | | Total blindness | 7 | |
| | | | Not testable due to lack of cooperation or a too severe clinical picture | 9 | |
| | | | Missing data | 99 | |
| | | Compensatory strategies | Not necessary* | 0 | |
| | | | Not testable for age (<2.5yo) | 00 | |
| | | | Normal (>7/10) (in this case sign as 'not necessary'* the next levels for near VA) | 1 | |
| | | | Near-normal (>3 and ≤7/10) | 2 | |
| | | | Mild low vision (> 2 and ≤3/10) | 3 | |
| | | | Moderate low vision (> 1 and ≤2/10) | 4 | |
| | | | Severe low vision (> 0.05 and ≤1/10) | 5 | |
| | | | Partial blindness (≤0.05/10) | 6 | |
| | | | Total blindness | 7 | |
| | | | Not testable due to lack of cooperation or a too severe clinical picture | 9 | |
| | | | Missing data | 99 | |

| | | | | | |
|---|---|---|---|---|---|
| Near Visual Acuity | Single Symbols Test | No compensatory strategies | Not necessary* | 0 | |
| | | | Not testable for age (<2.5yo) | 00 | |
| | | | Normal (>7/10) (in this case sign as 'not necessary'* the next levels for near VA) | 1 | |
| | | | Near-normal (>3 and ≤7/10) | 2 | |
| | | | Mild low vision (> 2 and ≤3/10) | 3 | |
| | | | Moderate low vision (> 1 and ≤2/10) | 4 | |
| | | | Severe low vision (> 0.05 and ≤1/10) | 5 | |
| | | | Partial blindness (≤0.05/10) | 6 | |
| | | | Total blindness | 7 | |

| | | | | | |
|---|---|---|---|---|---|
| | | | Not testable due to lack of cooperation or a too severe clinical picture | 9 | |
| | | | Missing data | 99 | |
| | | Compensatory strategies | Not necessary* | 0 | |
| | | | Not testable for age (<2.5yo) | 00 | |
| | | | Normal (>7/10) (in this case sign as ‘not necessary’* the next levels for near VA) | 1 | |
| | | | Near-normal (>3 and ≤7/10) | 2 | |
| | | | Mild low vision (> 2 and ≤3/10) | 3 | |
| | | | Moderate low vision (> 1 and ≤2/10) | 4 | |
| | | | Severe low vision (> 0.05 and ≤1/10) | 5 | |
| | | | Partial blindness (≤0.05/10) | 6 | |
| | | | Total blindness | 7 | |
| | | | Not testable due to lack of cooperation or a too severe clinical picture | 9 | |
| | | | Missing data | 99 | |

| | | | |
|---|---|---|---|
| Grating Acuity | Not necessary (if recognition VA is measurable) | 0 | |
| | Normal | 1 | |
| | Reduced (standard distance for age) | 2 | |
| | Reduced (only testable for lower distances compared to standard age) | 3 | |
| | Low vision card perception only/only response to the 0.50 cyc/deg paddle | 4 | |
| | No response | 5 | |
| | Not testable due to lack of cooperation or a too severe clinical picture | 9 | |
| | Missing data | 99 | |

**Visual Acuity Simmetry**

| | | | |
|---|---|---|---|
| Distance Visual Acuity | Not testable for age (<4yo) | 00 | |
| | Symmetric (multiple symbols/Line Tests) | 1 | |
| | Symmetric (single symbols test) | 2 | |
| | Not symmetric (difference > 1.5/10) | 3 | |
| | Not testable due to lack of cooperation or a too severe clinical picture | 9 | |
| | Missing data | 99 | |
| Near Visual Acuity | Not testable for age (<2.5yo) | 00 | |

| | | | |
|---|---|---|---|
| | Symmetric (multiple symbols/Line Tests) | 1 | |
| | Symmetric (single symbols test) | 2 | |
| | Not symmetric (difference > 1.5/10) | 3 | |
| | Not testable due to lack of cooperation or a too severe clinical picture | 9 | |
| | Missing data | 99 | |
| Grating Acuity | Not necessary | 0 | |
| | Symmetric | 1 | |
| | Not symmetric (difference > 2 cards or 2 paddles) | 2 | |
| | Not testable due to lack of cooperation or a too severe clinical picture | 9 | |
| | Missing data | 99 | |

**Contrast sensitivity (CS)**

| | | | | | |
|---|---|---|---|---|---|
| Distance Contrast sensitivity (3-5 meters) | Multiple Symbols (Line Tests) | No compensatory strategies | Not testable for age (<4yo) | 00 | |
| | | | Normal (in this case sign as ‘not necessary’* the next levels for distance CS) | 1 | |
| | | | Mild alteration | 2 | |
| | | | Moderate alteration | 3 | |
| | | | Severe alteration | 4 | |
| | | | No response | 5 | |
| | | | Not testable due to lack of cooperation or a too severe clinical picture | 9 | |
| | | | Missing data | 99 | |
| | | Compensatory strategies | Not necessary* | 0 | |
| | | | Not testable for age (<4yo) | 00 | |
| | | | Normal (in this case sign as ‘not necessary’* the next levels for distance CS) | 1 | |
| | | | Mild alteration | 2 | |
| | | | Moderate alteration | 3 | |
| | | | Severe alteration | 4 | |
| | | | No response | 5 | |
| | | | Not testable due to lack of cooperation or a too severe clinical picture | 9 | |
| | | | Missing data | 99 | |

| | | | | | |
|---|---|---|---|---|---|
| Distance Contrast sensitivity (3-5 meters) | Single Symbols Test | No compensatory strategies | Not necessary* | 0 | |
| | | | Not testable for age (<4yo) | 00 | |
| | | | Normal (in this case sign as 'not necessary'* the next levels for distance CS) | 1 | |
| | | | Mild alteration | 2 | |
| | | | Moderate alteration | 3 | |
| | | | Severe alteration | 4 | |
| | | | No response | 5 | |
| | | | Not testable due to lack of cooperation or a too severe clinical picture | 9 | |
| | | | Missing data | 99 | |
| | | Compensatory strategies | Not necessary* | 0 | |
| | | | Not testable for age (<4yo) | 00 | |
| | | | Normal | 1 | |
| | | | Mild alteration | 2 | |
| | | | Moderate alteration | 3 | |
| | | | Severe alteration | 4 | |
| | | | No response | 5 | |
| | | | Not testable due to lack of cooperation or a too severe clinical picture | 9 | |
| | | | Missing data | 99 | |

| | | | | | |
|---|---|---|---|---|---|
| Near Contrast sensitivity | Multiple Symbols (Line Tests) | No compensatory strategies | Not testable for age (<2.5yo) | 00 | |
| | | | Normal (in this case sign as 'not necessary'* the next levels for near CS) | 1 | |
| | | | Mild alteration | 2 | |
| | | | Moderate alteration | 3 | |
| | | | Severe alteration | 4 | |
| | | | No response | 5 | |
| | | | Not testable due to lack of cooperation or a too severe clinical picture | 9 | |

| | | | | | |
|---|---|---|---|---|---|
| | | | Missing data | 99 | |
| | | Compensatory strategies | Not necessary* | 0 | |
| | | | Not testable for age (<2.5yo) | 00 | |
| | | | Normal (in this case sign as 'not necessary'* the next levels for near CS) | 1 | |
| | | | Mild alteration | 2 | |
| | | | Moderate alteration | 3 | |
| | | | Severe alteration | 4 | |
| | | | No response | 5 | |
| | | | Not testable due to lack of cooperation or a too severe clinical picture | 9 | |
| | | | Missing data | 99 | |

| | | | | | |
|---|---|---|---|---|---|
| Near Contrast sensitivity | Single Symbols Test | No compensatory strategies | Not necessary* | 0 | |
| | | | Not testable for age (<2.5yo) | 00 | |
| | | | Normal (in this case sign as 'not necessary'* the next levels for near CS) | 1 | |
| | | | Mild alteration | 2 | |
| | | | Moderate alteration | 3 | |
| | | | Severe alteration | 4 | |
| | | | No response | 5 | |
| | | | Not testable due to lack of cooperation or a too severe clinical picture | 9 | |
| | | | Missing data | 99 | |
| | | Compensatory strategies | Not necessary* | 0 | |
| | | | Not testable for age (<2.5yo) | 00 | |
| | | | Normal | 1 | |
| | | | Mild alteration | 2 | |
| | | | Moderate alteration | 3 | |
| | | | Severe alteration | 4 | |
| | | | No response | 5 | |

| | | | | | |
|---|---|---|---|---|---|
| | | | Not testable due to lack of cooperation or a too severe clinical picture | 9 | |
| | | | Missing data | 99 | |

**Detection Test**

| | | |
|---|---|---|
| Not necessary (if Contrast Sensitivity Line test or Single Symbol Test have been administered) | 0 | |
| Normal | 1 | |
| Reduced | 2 | |
| Response only to full contrast stimulus | 3 | |
| No response | 4 | |
| Not testable due to lack of cooperation or a too severe clinical picture | 9 | |
| Missing data | 99 | |

**Visual Field**

| | | |
|---|---|---|
| Not testable for age (< 12yo) | 00 | |
| Normal | 1 | |
| Quadrantopsia/scotoma | 2 | |
| Hemianopsia | 3 | |
| Diffuse reduction | 4 | |
| Not testable due to lack of cooperation or a too severe clinical picture | 9 | |
| Missing data | 99 | |

**Visual Localization Areas**

| | | |
|---|---|---|
| Not necessary (if Visual Field can be studied) | 0 | |
| Normal | 1 | |
| Direction preference (Right/left/up/down) | 2 | |
| Diffuse alteration (with/without compensatory strategies) | 3 | |
| Only with environmental adaptation | 4 | |
| No response | 5 | |
| Not testable due to lack of cooperation or a too severe clinical picture | 9 | |
| Missing data | 99 | |

**Behavioural Response to Light**

| | | |
|---|---|---|
| Indifferent | 1 | |
| Photophobia | 2 | |
| Night blindness | 3 | |
| Not testable due to lack of cooperation or a too severe clinical picture | 9 | |
| Missing data | 99 | |

## SECTION 4 – OCULO-MOTOR ASPECTS

**Head Postural Behaviour**

| | | |
|---|---|---|
| Absent | 1 | |
| Incostant/variable | 2 | |
| Mild head tilt/turn/tremor | 3 | |
| Severe head tilt/turn/tremor | 4 | |
| Not testable due to a too severe clinical picture | 5 | |
| Missing data | 99 | |

**Eyelids**

| | | |
|---|---|---|
| Normal | 1 | |
| Unilateral mild to moderate ptosis | 2 | |
| Bilateral mild to moderate ptosis | 3 | |
| Unilateral severe ptosis (covering the eye pupil) | 4 | |
| Bilateral severe ptosis (covering the eye pupil) | 5 | |
| Not testable due to lack of cooperation | 9 | |
| Missing data | 99 | |

**Pupillary Statics**

| | | |
|---|---|---|
| Normal | 1 | |
| Altered (one eye) | 2 | |
| Altered (oboth eyes) | 3 | |
| Not testable due to lack of cooperation or a too severe clinical picture | 9 | |
| Missing data | 99 | |

**Pupillary Dinamics**

| | | |
|---|---|---|
| Normal | 1 | |
| Mild alteration | 2 | |
| Moderate-severe alteration | 3 | |
| Not testable due to lack of cooperation or a too severe clinical picture | 9 | |
| Missing data | 99 | |

**Ocular Motility**

| | | |
|---|---|---|
| Normal | 1 | |
| Mild hyperfunction/limitation | 2 | |
| Moderate-severe hyperfunction/limitation | 3 | |
| Paralytic limitation | 4 | |
| Not testable due to lack of cooperation or a too severe clinical picture | 9 | |
| Missing data | 99 | |

**Visual Axis Alignment**

| | | |
|---|---|---|
| Normal | 1 | |
| Mild misalignment with alternating fixation | 2 | |
| Severe misalignment without alternating fixation | 3 | |

| | | |
|---|---|---|
| Paralytic misalignment | 4 | |
| Not testable due to lack of cooperation or a too severe clinical picture | 9 | |
| Missing data | 99 | |

**Fixation**

| | | | |
|---|---|---|---|
| No compensatory strategies nor environmental adaptation (LEVEL A) | Stable, durable, binocular; no difference between near and distance | 1 | |
| | Durable, but not binocular and/or alternating and/or durable but slightly different from near and distance | 2 | |
| | Instable/slightly discontinuous and/or different from near and distance but sufficiently durable | 3 | |
| | Discontinuous | 4 | |
| | Fluctuating/eccentric | 5 | |
| | Sporadic | 6 | |
| | Absent | 7 | |
| | Not testable due to lack of cooperation or a too severe clinical picture | 9 | |
| | Missing data | 99 | |
| Compensatory strategies without environmental adaptation (LEVEL B) | Not necessary (already tested in the previous level) | 0 | |
| | Stable, durable, binocular; no difference between near and distance | 1 | |
| | Durable, but not binocular and/or alternating and/or durable but slightly different from near and distance | 2 | |
| | Instable/slightly discontinuous and/or different from near and distance but sufficiently durable | 3 | |
| | Discontinuous | 4 | |
| | Fluctuating/eccentric | 5 | |
| | Sporadic | 6 | |
| | Absent | 7 | |
| | Not testable due to lack of cooperation or a too severe clinical picture | 9 | |
| | Missing data | 99 | |
| Minor Environmental Adaptation (LEVEL C) | Not necessary (already tested in the previous level) | 0 | |
| | Stable, durable, binocular; no difference between near and distance | 1 | |
| | Durable, but not binocular and/or alternating and/or durable but slightly different from near and distance | 2 | |
| | Instable/slightly discontinuous and/or different from near and distance but sufficiently durable | 3 | |

| | | | |
|---|---|---|---|
| | Discontinuous | 4 | |
| | Fluctuating/eccentric | 5 | |
| | Sporadic | 6 | |
| | Absent | 7 | |
| | Not testable due to lack of cooperation or a too severe clinical picture | 9 | |
| | Missing data | 99 | |
| Major Environmental Adaptation (LEVEL D) | Not necessary (already tested in the previous level) | 0 | |
| | Stable, durable, binocular; no difference between near and distance | 1 | |
| | Durable, but not binocular and/or alternating and/or durable but slightly different from near and distance | 2 | |
| | Instable/slightly discontinuous and/or different from near and distance but sufficiently durable | 3 | |
| | Discontinuous | 4 | |
| | Fluctuating/eccentric | 5 | |
| | Sporadic | 6 | |
| | Absent | 7 | |
| | Not testable due to lack of cooperation or a too severe clinical picture | 9 | |
| | Missing data | 99 | |

**Smooth Pursuit**

| | | | |
|---|---|---|---|
| No compensatory strategies nor environmental adaptation (LEVEL A) | Durable, complete and binocular | 1 | |
| | Durable but incomplete/asymmetric/non binocular | 2 | |
| | Slightly discontinuous in all or great parts of directions | 3 | |
| | Discontinuous/jerky/augmented latency | 4 | |
| | Inconstant/eccentric/fragmented (continuous losses of fixation) | 5 | |
| | Possible only for small arc | 6 | |
| | Absent | 7 | |
| | Not testable due to lack of cooperation or a too severe clinical picture | 9 | |
| | Missing data | 99 | |
| Compensatory strategies without environmental adaptation | Not necessary (already tested in the previous level) | 0 | |
| | Durable, complete and binocular | 1 | |

| | | | |
|---|---|---|---|
| (LEVEL B) | Durable but incomplete/asymmetric/non binocular | 2 | |
| | Slightly discontinuous in all or great parts of directions | 3 | |
| | Discontinuous/jerky/augmented latency | 4 | |
| | Inconstant/eccentric/fragmented (continuous losses of fixation) | 5 | |
| | Possible only for small arc | 6 | |
| | Absent | 7 | |
| | Not testable due to lack of cooperation or a too severe clinical picture | 9 | |
| | Missing data | 99 | |
| Minor Environmental Adaptation (LEVEL C) | Not necessary (already tested in the previous level) | 0 | |
| | Durable, complete and binocular | 1 | |
| | Durable but incomplete/asymmetric/non binocular | 2 | |
| | Slightly discontinuous in all or great parts of directions | 3 | |
| | Discontinuous/jerky/augmented latency | 4 | |
| | Inconstant/eccentric/fragmented (continuous losses of fixation) | 5 | |
| | Possible only for small arc | 6 | |
| | Absent | 7 | |
| | Not testable due to lack of cooperation or a too severe clinical picture | 9 | |
| | Missing data | 99 | |
| Major Environmental Adaptation (LEVEL D) | Not necessary (already tested in the previous level) | 0 | |
| | Durable, complete and binocular | 1 | |
| | Durable but incomplete/asymmetric/non binocular | 2 | |
| | Slightly discontinuous in all or great parts of directions | 3 | |
| | Discontinuous/jerky/augmented latency | 4 | |
| | Inconstant/eccentric/fragmented (continuous losses of fixation) | 5 | |
| | Possible only for small arc | 6 | |
| | Absent | 7 | |
| | Not testable due to lack of cooperation or a too severe clinical picture | 9 | |
| | Missing data | 99 | |

**Saccades**

| | | | |
|---|---|---|---|
| No compensatory strategies nor environmental adaptation (LEVEL A) | Fluid, complete, normal latency, conjugacy and precision, no evident hypo – or hypermetria | 1 | |
| | Fluid, incomplete and/or asymmetric and/or not binocular | 2 | |
| | Slight alteration (hypo-hypermetria, fluidity, latency) | 3 | |
| | Moderate alteration (hypo-hypermetria, fluidity, latency) | 4 | |
| | Severe alteration (hypo-hypermetria, fluidity, latency) | 5 | |
| | Sporadic/difficult to elicit (conditioned by attention) | 6 | |
| | Absent | 7 | |
| | Not testable due to lack of cooperation or a too severe clinical picture | 9 | |
| | Missing data | 99 | |
| Compensatory strategies without environmental adaptation (LEVEL B) | Not necessary (already tested in the previous level) | 0 | |
| | Fluid, complete, normal latency, conjugacy and precision, no evident hypo – or hypermetria | 1 | |
| | Fluid, incomplete and/or asymmetric and/or not binocular | 2 | |
| | Slight alteration (hypo-hypermetria, fluidity, latency) | 3 | |
| | Moderate alteration (hypo-hypermetria, fluidity, latency) | 4 | |
| | Severe alteration (hypo-hypermetria, fluidity, latency) | 5 | |
| | Sporadic/difficult to elicit (conditioned by attention) | 6 | |
| | Absent | 7 | |
| | Not testable due to lack of cooperation or a too severe clinical picture | 9 | |
| | Missing data | 99 | |
| Minor Environmental Adaptation (LEVEL C) | Not necessary (already tested in the previous level) | 0 | |
| | Fluid, complete, normal latency, conjugacy and precision, no evident hypo – or hypermetria | 1 | |
| | Fluid, incomplete and/or asymmetric and/or not binocular | 2 | |
| | Slight alteration (hypo-hypermetria, fluidity, latency) | 3 | |
| | Moderate alteration (hypo-hypermetria, fluidity, latency) | 4 | |
| | Severe alteration (hypo-hypermetria, fluidity, | 5 | |

| | | | |
|---|---|---|---|
| | latency) | | |
| | Sporadic/difficult to elicit (conditioned by attention) | 6 | |
| | Absent | 7 | |
| | Not testable due to lack of cooperation or a too severe clinical picture | 9 | |
| | Missing data | 99 | |
| Major Environmental Adaptation (LEVEL D) | Not necessary (already tested in the previous level) | 0 | |
| | Fluid, complete, normal latency, conjugacy and precision, no evident hypo – or hypermetria | 1 | |
| | Fluid, incomplete and/or asymmetric and/or not binocular | 2 | |
| | Slight alteration (hypo-hypermetria, fluidity, latency) | 3 | |
| | Moderate alteration (hypo-hypermetria, fluidity, latency) | 4 | |
| | Severe alteration (hypo-hypermetria, fluidity, latency) | 5 | |
| | Sporadic/difficult to elicit (conditioned by attention) | 6 | |
| | Absent | 7 | |
| | Not testable due to lack of cooperation or a too severe clinical picture | 9 | |
| | Missing data | 99 | |

**Saccadic Exploration**

| | | | |
|---|---|---|---|
| No compensatory strategies nor environmental adaptation (LEVEL A) | Well organized and systematic | 1 | |
| | Mild alteration of explorative pattern organization | 2 | |
| | Moderate alteration of explorative pattern organization | 3 | |
| | Severe alteration of explorative pattern organization | 4 | |
| | Only possible for near distance and not functional | 5 | |
| | Absent | 6 | |
| | Not testable due to lack of cooperation or a too severe clinical picture | 9 | |
| | Missing data | 99 | |
| Compensatory strategies without environmental adaptation (LEVEL B) | Not necessary (already tested in the previous level) | 0 | |
| | Well organized and systematic | 1 | |
| | Mild alteration of explorative pattern organization | 2 | |
| | Moderate alteration of explorative pattern organization | 3 | |
| | Severe alteration of explorative pattern organization | 4 | |
| | Only possible for near distance and not functional | 5 | |
| | Absent | 6 | |
| | Not testable due to lack of cooperation or a too | 9 | |

| | | | |
|---|---|---|---|
| | severe clinical picture | | |
| | Missing data | 99 | |
| Minor Environmental Adaptation (LEVEL C) | Not necessary (already tested in the previous level) | 0 | |
| | Well organized and systematic | 1 | |
| | Mild alteration of explorative pattern organization | 2 | |
| | Moderate alteration of explorative pattern organization | 3 | |
| | Severe alteration of explorative pattern organization | 4 | |
| | Only possible for near distance and not functional | 5 | |
| | Absent | 6 | |
| | Not testable due to lack of cooperation or a too severe clinical picture | 9 | |
| | Missing data | 99 | |
| Major Environmental Adaptation (LEVEL D) | Not necessary (already tested in the previous level) | 0 | |
| | Well organized and systematic | 1 | |
| | Mild alteration of explorative pattern organization | 2 | |
| | Moderate alteration of explorative pattern organization | 3 | |
| | Severe alteration of explorative pattern organization | 4 | |
| | Only possible for near distance and not functional | 5 | |
| | Absent | 6 | |
| | Not testable due to lack of cooperation or a too severe clinical picture | 9 | |
| | Missing data | 99 | |

**Abnormal Ocular Movements**

| | | |
|---|---|---|
| Absent | 1 | |
| Inconstant | 2 | |
| Simple nystagmus (one direction), mild | 3 | |
| Simple nystagmus (one direction), moderate-severe | 4 | |
| Multi-directional nystagmus without not conjugated/erratic/deviation associated movements | 5 | |
| Multi-directional nystagmus with not conjugated/erratic/deviation associated movements | 6 | |
| Roving movements | 7 | |
| Not testable due to lack of cooperation or a too severe clinical picture | 9 | |
| Missing data | 99 | |

**Stereopsis**

| | | |
|---|---|---|
| Not testable for age (<6 mo) | 00 | |
| Present | 1 | |
| Partial | 2 | |
| Absent | 3 | |
| Not testable due to lack of cooperation or a too severe clinical picture | 9 | |
| Missing data | 99 | |
